# Study protocol of an open-label, single arm phase II trial investigating the efficacy and safety of Trifluridine/Tipiracil combined with irinotecan as a second line therapy in patients with cholangiocarcinoma (TRITICC)

**DOI:** 10.1186/s12885-023-10972-6

**Published:** 2023-05-22

**Authors:** Linde Kehmann, Marie-Luise Berres, Maria Gonzalez-Carmona, Dominik P. Modest, Raphael Mohr, Alexander Wree, Marino Venerito, Christian Strassburg, Verena Keitel, Christian Trautwein, Tom Luedde, Christoph Roderburg

**Affiliations:** 1 Department of Hepatology and Gastroenterology, Campus Virchow Klinikum, Charité University Medicine Berlin, 13353 Berlin, Germany; 2 Servier Deutschland GmbH, Elsenheimerstr, 53, 80687 München, Germany; 3 Medical Department III, University Hospital of Aachen, Aachen, 52074 Germany; 4 Department of Internal Medicine I, University Hospital of Bonn, Venusberg-Campus 1, 53127 Bonn, Germany; 5 Department of Hematology, Oncology and Tumorimmunology, Charité-Universitätsmedizin Berlin, Freie Universität Berlin, Humboldt-Universität Zu Berlin, and Berlin Institute of Health, 10117 Berlin, Germany; 6 Department of Gastroenterology, Hepatology & Infectious Diseases, Otto-Von-Guericke University Hospital, 39120 Magdeburg, Germany; 7 Clinic for Gastroenterology, Hepatology & Infectious Diseases, University Hospital of Düsseldorf, Medical Faculty of Heinrich Heine University Düsseldorf, Moorenstraße 5, 40225 Düsseldorf, Germany

**Keywords:** Chemotherapy, Biliary tract cancer, Cholangiocarcinoma, FTD/TPI (Trifluridine/Tipiracil), Gall bladder carcinoma, Irinotecan, Second-line treatment, Translational research

## Abstract

**Background:**

The prognosis of patients with advanced biliary tract cancer (BTC) who have progressed on gemcitabine plus cisplatin is dismal. Trifluridine/tipiracil (FTD/TPI) and irinotecan have proven efficacy in different gastrointestinal malignancies. We therefore hypothesized that this combination might improve the therapeutic outcome in patients with BTC after failure of first line treatment.

**Methods:**

TRITICC is an interventional, prospective, open-label, non-randomised, exploratory, multicentre, single-arm phase IIA clinical trial done in 6 sites with expertise in managing biliary tract cancer across Germany. A total of 28 adult patients (aged ≥ 18 years) with histologically verified locally advanced or metastatic biliary tract cancer (including cholangiocarcinoma and gallbladder or ampullary carcinoma) with documented radiological disease progression to first-line gemcitabine based chemotherapy will be included to receive a combination of FTD/TPI plus irinotecan according to previously published protocols. Study treatment will be continued until disease progression according to RECIST 1.1 criteria or occurrence of unacceptable toxicity. The effect of FTD/TPI plus irinotecan on progression-free survival will be analyzed as primary endpoint. Safety (according to NCI-CTCAE), response rates and overall survival are secondary endpoints. In addition, a comprehensive translational research program is part of the study and might provide findings about predictive markers with regard to response, survival periods and resistance to treatment.

**Discussion:**

The aim of TRITICC is to evaluate the safety and efficacy of FTD/TPI plus irinotecan in patients with biliary tract cancer refractory to previous Gemcitabine based treatment.

**Trial registration:**

EudraCT 2018–002936-26; NCT04059562

## Background

Biliary tract cancer (BTC) represents a heterogeneous group of malignant tumours that may arise at any point of the biliary tree [1]. Despite being rare tumors, the global incidence of BTC is rising, currently accounting for ~ 15% of all primary liver cancers and ~ 3% of gastrointestinal malignancies [1]. In Germany about 5,000 BTC are newly diagnosed per year [2, 3]. The “silent” presentation of BTC combined with their highly aggressive nature and refractoriness to chemotherapy contribute to their alarming mortality, representing ~ 2% of all cancer-related deaths worldwide yearly [1].

The ABC-02 study established cisplatin and gemcitabine as first-line treatment in patients with advanced biliary tract cancer and represented the standard of care for many years [4]. Recently, the TOPAZ-1 trial demonstrated improved overall survival with durvalumab in combination with cisplatin and gemcitabine versus chemotherapy alone [5]. However, after failure of first-line treatment, many patients experience a rapid decline in performance status and only 15–25% receive second-line chemotherapy [6, 7]. Therefore, the role of second-line cytotoxic chemotherapy after progression on cisplatin and gemcitabine has remained unclear until now. A systematic review and meta-analysis of phase II data recently provided evidence for second-line chemotherapy to prolong median progression-free (PFS) and overall survival (OS) [8]. Walter and colleagues provided strong evidence that the efficacy of combination therapies is significantly higher compared to monotherapies in BTC-patients refractory to a first line therapy [6]. In a phase-3 study, second-line FOLFOX versus active symptom control alone demonstrated a moderate but significant improvement of survival and might be regarded as a standard of care [9] A potential alternative might be the addition of irinotecan or nanoliposomal pegylated irinotecan to fluoropyrimidines—a combination that also improved survival in a randomized Korean study [10]. Therefore, it might be concluded that available second-line evidence comprises doublet regimens with platinum and irinotecan, both in combination with fluoropyrimidines that are based on limited evidence and questionable generalizability. Thus, there is a high need for novel treatment concepts in patients without targetable genetic alterations [11].

Trifluridine/tipiracil (Lonsurf(®)) is a novel, orally active, antimetabolite agent comprised of trifluridine, a thymidine-based nucleoside analogue, and tipiracil, a potent thymidine phosphorylase inhibitor. Trifluridine is incorporated into DNA via phosphorylation, ultimately inhibiting cell proliferation. Tipiracil increases systemic exposure of trifluridine when coadministered [12].Trifluridine/tipiracil has recently been approved for the treatment of adult patients with metastatic colorectal cancer (mCRC) who are refractory to, or are not considered candidates for, current standard chemotherapy [13] and in gastric cancer after failure of standard treatments [14]. Notably, FTD/TPI proved efficacy in patients with 5-FU resistance [13, 15]. Recently, in a nicely conducted phase II study, FTD/TPI demonstrated promising antitumor activity with acceptable toxicity in heavily pretreated patients with advanced BTC [16]. Moreover, in a recently published single arm phase II trial comprising 28 patients (27 evaluable) with advanced BTC, the combination of trifluridine/tipiracil plus irinotecan showed promising safety and activity (ORR 20% and DCR 45%, 16-week PFS 37% and median OS 52,6 weeks, respectively) [17]. Moreover, in a recent randomized trial analyzing a total of 120 patients it turned out that mFOLFIRI was tolerable and showed comparable efficacy to mFOLFOX in the second-line treatment of BTC [18], supporting a deeper analysis of the combination of trifluridine/tipiracil plus irinotecan in further clinical trials.

## Objectives and endpoints

### Objectives

The primary objective of the TRITICC trial is to examine the efficacy of a combination therapy of FTD/TPI and Irinotecan in patients with advanced, non resectable or metastatic BTC after failure to respond to a previous gemcitabine based treatment. Secondary objectives are to determine the efficacy and safety of the treatment concept, health-related quality of life (HR-QoL) and translational investigation.

### Endpoints

The primary endpoint is median progression free survival (PFS) assessed by the local investigator. Secondary endpoints are Progression-free survival rate @ 4 months, median overall survival and proportion of patients with an objective response according to RECIST 1.1 [19]. Secondary safety endpoints are type, grade, frequency and severity of adverse events according NCI CTCAE version 5.0. Secondary endpoint quality of life will be assessed by EORTC QLQ-30 and the EQ-5D-5Lquestionnaires. Secondary translational endpoints are cfDNA exome sequencing, circulating miRNAs and lncRNAs, concentrations of inflammatory cytokines and gut microbiome analysis. (Table 1).

**Table 1:** Objectives and endpoints of the TRITICC trial

**1. Objectives**
**1.1 Primary Objectives** • To assess the efficacy of a combination therapy of FTD/TPI and Irinotecan in patients with advanced, non resectable or metastatic cholangio- and gallbladder carcinoma after failure to respond to a previous gemcitabine treatment.
**1.2 Secondary Objectives** • To determine efficacy and safety of the treatment concept • To determine health-related quality of life (HR-QoL) • To determine translational observations (circulating miRNAs and lncRNAs, concentrations of inflammatory cytokines, gut microbiome analysis)
**2. Endpoints**
**2.1 Primary Endpoint** • Median progression free survival (PFS) assessed by the local investigator
**2.2 Secondary Endpoints**
**Efficacy** • Progression-free survival rate @ 4 months defined as the proportion of patients with non-progressive disease 4 months after inclusion by intention to treat analysis • Median overall survival • Proportion of patients with an objective response according to RECIST 1.1
**Safety** **• Type, frequency and severity of adverse events with severity according to NCI CTCAE version**
**Health-Related Quality of Life** • HR-QoL according to EORTC QLQ-C30 and the EQ-5D-5L
**Translational part** • **cfDNA exome sequencing**: a panel of 28 mutations (e.g. IDH1, IDH2, SMAD4) that are specific for BTC will be recapitulated in patient’s serum by using cfDNA exome sequencing. • **Circulating miRNAs and lncRNAs**: Alterations in serum miRNAs as prognostic markers will be analysed using array based techniques in representative patients and significantly altered miRNAs will be validated using qPCR in the whole cohort of patients. • Concentrations of inflammatory cytokines: A panel of inflammatory cytokines will be analyzed using FACS bead technology in the serum of patients. This panel will include analysis of Osteopontin, suPAR, APRIL, TWEAK, TRAIL, CCL1, 2, 5, and CXCR1, 2, 5, 9) • **Gut microbiome analysis:** By using high-throughput sequencing of bacterial 16S RNA a complete and unbiased analysis of the gut microbiota will be performed in each individual patient. Both the baseline composition and alterations during therapy will be correlated with PFS and response to therapy.

## Methods/study design

TRITICC is an interventional, prospective, open-label, non-randomised, exploratory, multicentre, single-arm phase IIA clinical trial organized and sponsored by the University of Dusseldorf. Eligible patients with histologically or cytologically confirmed, non-resectable, locally advanced or metastatic cholangiocarcinoma or gall bladder carcinoma will receive a second line combination chemotherapy with FTD/TPI and irinotecan in cycles of 14 days. In total 28 patients (including 3 calculated dropouts and invalid cases) after failure of a gemcitabine based first-line therapy will be enrolled at up to 6 centers. Screening is expected to be necessary in 40 patients.

### Study duration

The recruitment of patients for the inclusion of the study was started in Q3 2021. The recruitment period will last 18 months. Patients will be treated until radiological progression. In average this is estimated being about 4 months. A follow up is planned every 3 months up to 6 months. Last patient last visit is anticipated for August 2023. Based on these assumptions, duration will be in total 40 months including 12 months evaluation and clinical study report.

### Trial population

Patients with cholangiocarcinoma or gall bladder carcinoma after progressing on a gemcitabine based first-line therapy and fulfilling the other inclusion criteria will be eligible to participate in this clinical trial.

### Inclusion criteria

1. Written informed consent incl. participation in translational research and any locally-required authorization (EU Data Privacy Directive in the EU) prior to performing any protocol-related procedures, including screening evaluations
2. Age ≥ 18 years at time of study entry
3. Histologically or cytologically confirmed, non-resectable, locally advanced or metastatic cholangiocarcinoma or gall bladder carcinoma
4. Measurable or assessable disease according to RECIST
5. Documented disease progression after prior gemcitabine or gemcitabine containing therapy. Examples of permitted therapies include, but are not limited to: a) Single agent gemcitabine); b) Any gemcitabine-based regimen, with or without maintenance gemcitabine
6. ECOG performance status 0–1
7. Ability to take medications orally
8. Adequate blood count, liver-enzymes, and renal function:
9. ANC > 1,500 cells/μL without the use of hematopoietic growth factors; and Platelet count ≥ 100 × 109/L (> 100,000 per mm3) and Hemoglobin > 9 g/dL (blood transfusions are permitted for patients with hemoglobin levels below 9 g/dL)
10. Serum total bilirubin ≤ 1.5 × upper normal limit (ULN) (biliary drainage is allowed for biliary obstruction; elevated bilirubin should be caused by obstruction not impaired liver function as assessed by albumin and INR values):
11. Albumin levels ≥ 3.0 g/dL
12. Patients not receiving therapeutic anticoagulation must have an INR < 1.5 ULN and PTT < 1.5 ULN within 7 days prior to inclusion. The use of full dose anticoagulants is allowed as long as the INR or PTT is within therapeutic limits (according to the medical standard in the institution) and the patient has been on a stable dose for anticoagulants for at least three weeks at the time of inclusion.
13. AST (SGOT) and ALT (SGPT) ≤ 5 × institutional upper limit of normal
14. Serum Creatinine ≤ 1.5 × ULN and a calculated glomerular filtration rate ≥ 30 mL per minute Adequate renal and bone marrow function
15. In case of liver cirrhosis: Child–Pugh A
16. Women of childbearing potential must have a negative pregnancy test and must agree to adequate birth control if conception is possible. Males must agree to adequate birth control

### Exclusion criteria

1. Age < 18 years
2. CNS metastases
3. Active, uncontrolled infection
4. Additional malignancy within the past 2 years (except adequately treated in-situ carcinoma of the cervix or non-melanoma skin cancer)
5. Clinically significant gastrointestinal disorders including bleeding, inflammation, occlusion, or diarrhea > grade 1
6. Any condition or comorbidity that, in the opinion of the investigator, would interfere with evaluation of study treatment or interpretation of patient safety or study results
7. Known hypersensitivity to FTD/TPI or Irinotecan or their components
8. Medication that is known to interfere with any of the agents applied in the trial
9. Pregnancy or lactating female
10. Prior partial or total gastrectomy
11. Previous radio- or radiochemotherapy, previous transarterial chemoembolisation (TACE), radiofrequencyablation (RFA) or selective intraarterial radiotherapy (SIRT) within 3 months prior to inclusion (except radiation for bone metastases)
12. Patients who might be dependent on the sponsor, site or the investigator
13. Patients who have been incarcerated or involuntarily institutionalized by court order or by the authorities § 40 Abs. 1 S. 3 Nr. 4 AMG.
14. Patients who are unable to consent because they do not understand the nature, significance and implications of the clinical trial and therefore cannot form a rational intention in the light of the facts [§ 40 Abs. 1 S. 3 Nr. 3a AMG].
15. Use of other investigational treatment within 5 half-lives of enrollment is prohibited.

### Gender and age selection

Adults of all genders equal or above 18 years are eligible for this clinical trial.

### Tumor imaging

For tumor assessment according to RECIST version 1.1 whenever possible, MRI should be chosen as examination method. However, conventional CT is permitted as alternative. The examination method once selected is to be retained for all examinations within the context of the study. Chest CT and additional skeletal scintigraphy /X-ray images of all affected bones in case of suspected bone metastases and additional cranial MRI or CT (MRI preferred whenever possible) in case of suspected CNS metastases are to be implemented. Additional imaging may be required in symptomatic patients if clinically indicated.

### Treatment regimen

Dosage and schedule were selected according to the results of Phase I study of FTD/TPI Plus Irinotecan and Bevacizumab in Advanced Gastrointestinal Tumors (Varghese AM et al.) [20].

FTD/TPI will be administered at a dose of 25 mg/m^2^ / dose twice daily on days 1–5 followed by a 9-days recovery period from day 6 trough day 14 of each 14-days treatment cycle.

The dosage is calculated according to body surface area (BSA) as displayed in Table 2. The dosage must not exceed 60 mg/dose. Table 2 also displays the necessary number of 15 mg and 20 mg tablets Lonsurf® to be taken. If any doses are missed, these doses must not be made up by the patient.

**Table 2:** Calculation of dosing for FTD/TPI in mg according to BSA used in the TRITICC trial

**Starting dose** **25 mg/m**^**2**^	**BSA (m**^**2**^**)**	**Dose in mg** **2 × daily**	**Tablets per dose**		**Total daily dose (mg)**
			**15 mg**	**20 mg**	
	< 1.10	25!	2!	1!	50!
	1.10 – 1.29	30	2	0	60
	1.30 – 1.49	35	1	1	70
	1.50 – 1.69	40	0	2	80
	1.70 – 1.89	45	3	0	90
	1.90 – 2.09	50	2	1	100
	2.10 – 2.29	55	1	2	110
	> 2.30	60	0	3	120

(!) At a total daily dose of 50 mg, patients should take 1 × 20 mg tablet in the morning and 2 × 15 mg tablets in the evening

Irinotecan will be administered at the same time as FTD/TPI on day 1 of each cycle at a dose of 180 mg/m^2^ / dose.

Treatment will be administered until radiological progress or inacceptable toxicity.

#### Dose modifications and dose delays

Toxicity is classified according to NCI-CTCAE version 5.0; the dose modifications are made based on the grading given in the latter. The treatment must not be modified if adverse reactions occur which, according to the investigator's estimation, do not result in severe or life-threatening consequences (e.g. alopecia). Should several different toxicities appear simultaneously, the respective greatest dose reduction decrement should be applied. If an adverse reaction is unequivocally attributable to only one of the cytostatic agents the dosages of the other substances do not need to be modified. A further dose escalation is not permitted.

In case permanent discontinuation or treatment interruption of more than two cycles of one of the substances is required due to toxicity, study treatment has to be terminated. The further treatment will be at the treating physician's discretion.

#### Dose modification of FTD/TPI

A maximum of 1 dose reduction level (20 mg/m^2^ BSA BID) is permitted for FTD/TPI. Table 3 displays the dose in mg to be taken twice daily calculated per m^2^ BSA. (Tables 4 and 5).

**Table 3:** Calculation of dose reduction for FTD/TPI in mg according to BSA used in the TRITICC trial

**Starting dose** **20 mg/m**^**2**^	**BSA (m**^**2**^**)**	**Dose in mg** **2 × daily**	**Tablets per dose**		**Total daily dose (mg)**
			**15 mg**	**20 mg**	
	< 1.14	20	0	1	40
	1.15 – 1.34	25!	2!	1!	50!
	1.35 – 1.59	30	2	0	60
	1.60 – 1.94	35	1	1	70
	1.95 – 2.09	40	0	2	80
	2.10 – 2.34	45	3	0	90
	> 2.35	50	2	1	100

(!) At a total daily dose of 50 mg, patients should take 1 × 20 mg tablet in the morning and 2 × 15 mg tablets in the evening

**Table 4:** Dose interruption and resumption criteria for haematological toxicities related to myelosuppression [21]

**Parameter**	**Interruption**	**Resumption criteria**^**a**^
Neutrophils	< 0.5 × 10^9^/L	< 1.5 × 10^9^/L
Platelets	< 50 × 10^9^/L	> 75 × 10^9^/L

^a^Resumption criteria applied to the start of the next cycle for all patients regardless of whether or not the interruption criteria were met

**Table 5:** Recommended dose modifications for FTF/TPI in case of haematological and non-haematological adverse reactions [21]

**Adverse reaction**	**Recommended dose modifications**
• Febrile neutropenia	• Interrupt dosing until toxicity resolves to Grade 1 or baseline
• Grade 4 neutropenia (< 0.5 × 10^9^/L) or thrombocytopenia (< 25 × 10^9^/L) that results in more than 1 week’s delay in start of next cycle	• When resuming dosing, decrease the dose level by 5 mg/m^2^/dose from the previous dose level
• CTCAE version 4 non-haematologic Grade 3 or Grade 4 adverse reaction; except for Grade 3 nausea and/or vomiting controlled by antiemetic therapy or diarrhoea responsive to antidiarrhoeal medicinal products	• Dose reductions are permitted to a minimum dose of 20 mg/m^2^/dose BID daily • Do not increase dose after it has been reduced

Dose escalation of FTD/TPI is not permitted after it has been reduced.

#### Dose modification of irinotecan

Table 6 displays recommended dose modifications for Irinotecan in case of haematological or non-haematological adverse reactions.

**Table 6:** Recommended dose interruption/ modification criteria

	**Grade 2**	**Grade 3**	**Grade 4**
1st occurence	No dose recuction, prophylaxis if possible	75% of the initial dose, prophylaxis if possible	Permanent discontinuation, unless continuation of treatment is in the patients best interest; if so 50% of the inizial dose following consultation with the study coordination centre
2nd occurence	75% of the initial dose	50% of the initial dose	
3rd occurence	50% of the initial dose	Definitive withdrawal, unless continuation of treatment is in the patients best interest	
4th occurence	Permanent discontinuation, unless continuation of treatment is in the patients best interest		

#### Concomitant medication

All medication, which is considered necessary for the patient’s welfare, and which is not expected to interfere with the evaluation of FTD/TPI, may be given at the discretion of the investigator. All concomitant medications must be recorded in the patient’s source documentation, as well as in the appropriate pages of the eCRF. Subjects should receive full supportive care, including transfusions of blood and blood products, anti-infective drugs, and antiemetics when appropriate.

All patients should be closely monitored for side effects of all concomitant medications regardless of the path of elimination.

#### Antiemetics

During the treatment with FTD/TPI and Irinotecan, owing a moderate emetic potential, an antiemetic treatment should be administered according to center-specific protocols using, among others, dexamethasone and 5-HT3 antagonists. In case of insufficient effect, aprepitant (Emend®) may be considered. Appropriate prophylactic antiemesis is recommended.

#### Anticoagulants

The use of full dose anticoagulants is allowed as long as the INR or PTT is within therapeutic limits (according to the medical standards in the institution) and the patient has been on a stable dose for anticoagulants for at least two weeks at the time of registration. During treatment close monitoring of INR for oral anticoagulants is recommended.

#### Growth factors

Hematopoietic growth factors (i.e., G-CSF) may be used according to institutional guidelines to treat febrile neutropenia, but should not be used as primary or secondary prophylaxis. Growth factors must be discontinued at least 48 h prior to initiation of the next treatment of chemotherapy.

#### Prohibited concomitant medication

The following medications/treatments are not allowed during the study participation, unless the patient is in the follow-up phase and has permanently discontinued any study medication:

Any investigational medicinal product or experimental therapy or participation in another clinical trial. Any other anti-tumor treatment including chemotherapy, targeted therapy, antibodies, any antihormonal tumor treatment, and immunotherapy other than FTD/TPI and Irinotecan.

Current data in gastric cancer indicate a potential worse survival for patients receiving fluoropyrimidine-derivates with the concomitant use of protone-pump inhibitors, which should be avoided if possible. For details of contraindications, Investigators should refer to the respective SmPC. Caution is required when using medicinal products that are human thymidine kinase substrates, e.g., zidovudine. Such medicinal products, if used concomitantly with FTD/TPI, may compete with the effector, trifluridine, for activation via thymidine kinases. Therefore, when using antiviral medicinal products that are human thymidine kinase substrates, monitor for possible decreased efficacy of the antiviral medicinal product, and consider switching to an alternative antiviral medicinal product that is not a human thymidine kinase substrate, such as lamivudine, and abacavir. It is unknown whether FTD/TPI may reduce the effectiveness of hormonal contraceptives. Therefore, women using hormonal contraceptive must also use a barrier contraceptive method.

### Statistical considerations

The primary endpoint, time till progression-free survival, will be analyzed using a non-parametric survival estimate. To this end the method of Turnbull will be used to compute the survival curve [22]. The variance of the survival will be estimated using the Greenwood formula. The log transformation will be used for the calculation of confidence intervals.

An exploratory analysis of secondary outcomes will be performed. Overall survival will be analyzed using the Kaplan–Meier estimate of survival. For the secondary outcomes, objective response and occurrence of adverse events the proportion of patients with the outcome and confidence intervals using the Wilson method will be calculated [23].

The statistical analysis will be carried out by the Coordination Center for Clinical Trials Düsseldorf (KKSD).

### Sample size determination

Sample size consideration for this study is based on the precision of the survival estimate for the outcome progression-free survival. Based on previous results with the combination of Irinotecan and 5-FU therapy in patients with biliary tract cancers we assume a median survival time of 3 months. Assuming an exponential failure time with a median survival of 3 months (hazard rate: 0.23 per month) and a censoring rate of 0.04 per month, a total sample size of 28 patients (including 3 calculated drop outs and invalid cases) results in an expected standard error of 0.09 for the 3 months survival.

### Assessment of severity/intensity of adverse events

The severity of any (S)AE's occurring will be assessed using the National Cancer Institute Common Terminology Criteria for Adverse Events (NCI-CTCAE), version 5.0.

Personnel of the trial center that is involved with the study must have access to a copy of the NCI-CTCAE version 5.0.

AE's are to be documented with the following details:

- Indication of the diagnosis or syndrome, duration of the AE, severity, assessment of whether an AE is serious or not (if an AE is to be classified as a SAE, additional reporting as SAE is required), assessment of the causal relationship with the study medication and the outcome of the AE's.
- In this study, all adverse events occurring from the time of signed informed consent by the patient until 30 days after the last administration of the study medication are to be documented as adverse events.
- A serious adverse event (SAE) or serious adverse drug reaction (SADR) is any AE that fulfils at least one of the following criteria: results in death, is life-threatening, requires inpatient hospitalization or prolongation of existing hospitalization, is a congenital anomaly/birth defect or constitutes an important medical event.
- Important medical events are defined as those occurrences that may not be immediately life threatening or result in death, hospitalization, or disability, but may jeopardize the subject or require medical or surgical intervention to prevent one of the other outcomes listed above. Medical and scientific judgment should be exercised in deciding whether such an AE should be considered serious. SAE’s must be reported immediately within 24 h knowledge of the event.

### Translational research

It is intended to collect plasma and serum samples from all patients, following separate declaration of consent. Samples are to be taken at baseline, at each restaging visit and at the end of the treatment visit. After centrifugation, the serum and plasma have to be stored at -80° Celsius for further analyses. The aim is to measure concentrations of inflammatory cytokines using FACS bead technology in the serum of patients. This panel will include analysis of Osteopontin, suPAR, APRIL, TWEAK, TRAIL, CCL1, 2, 5, and CXCR1, 2, 5, 9, which all have been demonstrated being regulated in the serum of patients with gastrointestinal cancers. Moreover concentrations of circulating miRNAs and lncRNAs will be measured in these samples. The ultimate aim of these retrospective analyses is to study the predictive value with regard to response, survival periods and development of resistance.

Liquid biopsies will be taken to perform translational research on cell-free DNA in blood, following separate declaration of consent. cfDNA exome sequencing will be performed using a panel of 28 mutations (e.g. IDH1, IDH2, SMAD4). These mutations will be recapitulated in patients´ serum by using cfDNA exome sequencing and correlated with clinical response, survival and development of resistance.

Stool analyses will be collected at baseline, at each restaging visit and at the end of the study treatment, following separate declaration of consent. In order to analyze the gut macrobiota we will perform different molecular-genetic examinations. First stool DNA will be prepared and bacterial gene sequences coding for 16S RNA will be quantified. As a screening examination a quantitative PCR using group-specific primer against variable parts of bacterial 16S RNA will be used. Next we will use a defined panel of 15 primer-pairs against the most prominent species of the gut microbiota to analyze rifaximin dependent alterations. Moreover it is planned to apply the highly innovative high-throughput sequencing of bacterial 16S RNA for complete and unbiased analysis of the gut microbiota.

### QoL assessment

Quality of life analysis will be performed by means of the EORTC QLQ-C30 [24–28] and the EQ-5D-5L- questionnaires [29]. The EORTC QLQ-C30 was developed as an instrument to measure patient-reported health-related quality of life. The current version 3.0 of the EORTC QLQ-C30 consists of 30 questions. It incorporates nine multi-item scales: five functional scales (physical, role, cognitive, emotional, and social); three symptom scales (fatigue, pain, and nausea/vomiting); and a global health and quality-of-life scale. Several single-item symptom measures are also included (dypnoea, insomnia, decreased appetite, constipation, diarrhea and financial difficulties due to physical condition/medical treatment).

EQ-5D is a standardized measure of health status developed by the EuroQol Group. The EQ-5D-5L essentially consists of the EQ-5D descriptive system and the EQ visual analogue scale (EQ VAS). The EQ-5D-5L descriptive system comprises the following 5 dimensions: mobility, self-care, usual activities, pain/discomfort and anxiety/depression. Each dimension has 5 levels: no problems, slight problems, moderate problems, severe problems, and extreme problems. The respondent is asked to indicate his/her health state by ticking (or placing a cross) in the box against the most appropriate statement in each of the 5 dimensions. This decision results in a 1-digit number expressing the level selected for that dimension. The digits for 5 dimensions can be combined in a 5-digit number describing the respondent’s health state. It should be noted that the numerals 1–5 have no arithmetic properties and should not be used as a cardinal score [29].

The EQ VAS records the respondent’s self-rated health on a 20 cm vertical, visual analogue scale (VAS) where the endpoints are labelled ‘the best health you can imagine’ and ‘the worst health you can imagine’. This information can be used as a quantitative measure of health outcome as judged by the individual respondents. Respondents are asked to simply ‘mark an X on the scale to indicate how your health is TODAY’ and then to ‘write the number you marked on the scale in the box below [29].

Results from the EQ-5D-5L descriptive system will be presented as health profile and index values. EQ VAS scores will be presented as mean values with standard deviation.

The EORTC QLQ-C30 and EQ-5D-5L questionnaires as paper version will be handed out to and completed by the patient, independently of the study personnel, on Day 1 of the first cycle visit prior to other study measures. Moreover the questionnaires will be handed out to and completed by the patient before each following cycle. The investigator should explain to the patient how to complete the questionnaires but neither he/she nor the research staff can assist the patient with their completion. The patient should complete the questionnaires by him/herself in a quiet place.

EORTC QLQ-C30 and EQ-5D-5L questionnaires filled in by the patient at the trial center have to be filed together with the patient’s medical file until the end of study visit in the trial center.

### Ethical considerations and trial registration

The protocol V03F from 09.08.2021 was developed and approved by the sponsor. The study will be performed in accordance with the ethical principles that have their origin in the Declaration of Helsinki and the ICH Good Clinical Practice guidelines. The local Ethics Committee of the University of Dusseldorf and the local ethic committees of the participating centers throughout Germany approved the study.

Any change in the study protocol and/or informed consent form will be presented to the named Ethics Committee. They have to be approved by the Ethics Committee before implementation (except for changes in logistics and administration or when necessary to eliminate immediate hazards). The sponsor, the competent authorities and the ethics committee may stop the clinical trial or participation of a clinical trial site in the clinical trial for medical, safety, regulatory, administrative or other reasons consistent with ICH-GCP, the respective European Union’s and national legislation. The study is registered at the ‘European Union Drug Regulating Authorities Clinical Trials’ (EudraCT 2018–002936-26) and clinicalstrials.gov (NCT04059562).

## Discussion

Biliary tract cancers are detected late in many patients due to the lack of typical symptoms, have a high mortality and limited treatment options. Although considered being a rare type of cancer, BTC is the second most common primary hepatic malignancy, accounting for approximately 20% of deaths from hepatobiliary cancers, which cause 13% of the total cancer mortality worldwide [3]. The global incidence of biliary tract cancers shows an overall increase; in Germany about 5000 BTC are newly diagnosed per year [2, 3]. In non resectable/ metastasized BTC, systemic first-line chemotherapy with Gemcitabin and Cisplatin represents the standard of care. In these patients an overall survival of 11.7 months can be achieved [7, 30]. The addition of check-point inhibitors might further increase overall survival to 12,8 months [5].

The usefulness of second-line therapy in patients refractory to gemcitabine-based first line chemotherapy in patients with BTC has long been the subject of controversy. Retrospective analyses have shown that patients in good general condition can benefit from cytotoxic chemotherapy in terms of overall survival and that combination therapies are more effective than single agents [6]. At present 5-FU based therapies are used in most patients [8]. Oxaliplatin in combination with 5-FU (FOLFOX) has proven efficacy in a recent phase-3 trial. However, the benefit achieved with in terms of median overall survival was marginal versus a purely symptomatic treatment strategy [31]. Besides Oxaliplatin, irinotecan-based treatments (e.g. FOLFIRI) have shown benefit as second line treatment of BTC [18, 32] Irinotecan (CPT-11) is an established substance in the therapy of pancreatico-biliary cancers. While the antitumor effect of Irinotecan monotherapy is low [33], the combination of Irinotecan and 5-FU (e.g. FUFIRI or FOLFIRI) has demonstrated efficacy in patients with BTC [21, 34], also when used as salvage [35].

Recently Liposomal irinotecan plus fluorouracil and leucovorin showed superior efficacy than fluorouracil and leucovorin in patients metastatic biliary tract cancer after progression on gemcitabine plus cisplatin [10]. Moreover, Irinotecan based therapies have demonstrated efficacy in terms of prolonged overall survival, progression-free survival and response rate in patients with metastatic pancreatic cancer after failure of gemcitabine treatment [21, 36, 37]. Together, these data provide a rational for potential efficacy of Irinotecan in salvage therapy of BTC as well.

Trifluridine/tipiracil (FTD/TPI, trade name Lonsurf®) [38], which was approved by the U.S. Food and Drug Administration on September 22^nd^, 2015 and by the European Medicines Agency on April 25^th^, 2016 represents a modern fluoropyrimidine derivate [39–41]. FTD/TPI withholds several pharmaco-dynamic and pharmacokinetic advantages compared to 5-FU and might help to overcome some of the limitations characteristic for 5-FU based therapies. First, FTD/TPI is administered orally and no permanent port catheter needs to be implanted before therapy. Second, the toxicity of FTD/TPI has been shown to be more favorable than that of 5-FU. Thus FTD/TPI bears unique characteristics that are not provided by other substances/ substance combinations currently used or tested in the context of BTC. The combination of FTD/TPI and Irinotecan has been proven efficacious and safe in different phase 1 trials [17, 42, 43], however, only few data on its potential in the second-line therapy of patients with advanced, non-resectable and metastatic adenocarcinoma of the biliary tract are available [17].

### Sample size calculation

Sample size consideration for this study is based on the precision of the survival estimate for In total 28 patients (including 3 calculated drop outs and invalid cases) with advanced biliary tract cancers after failure of a gemcitabine based first-line therapy will be enrolled the outcome progression-free survival. The primary endpoint, time till progression-free survival will be analyzed using a non-parametric survival estimate. To this end the method of Turnbull [22] will be used to compute the survival curve. The variance of the survival will be estimated using the Greenwood formula. The log transformation will be used for the calculation of confidence intervals. An exploratory analysis of secondary outcomes will be performed. Overall survival will be analyzed using the Kaplan–Meier estimate of survival. For the secondary outcomes, objective response and occurrence of adverse events the proportion of patients with the outcome and confidence intervals using the Wilson method will be calculated [23].

### Research hypothesis

We hypothesize that, based on the available results on 5-FU, the use of a combination therapy of FTD/TPI and Irinotecan might demonstrate anti-tumor activity in patients with advanced, non resectable or metastatic cholangio- or gallbladder carcinoma after failure to respond to a previous gemcitabine treatment.

## Data Availability

N/A.

## Abbreviations

BTC: Biliary tract cancer
FTD/TPI: Trifluridine/Tipiracil
CCA: Cholangiocellular carcinoma
TRITICC: Efficacy and safety of Trifluridine/Tipiracil in combination with Irinotecan as asecond line therapy in patients with cholangiocarcinoma
RECIST: Response Evaluation Criteria in Solid Tumours
NCI-CTCAE: National Cancer Institute Common Toxicity Criteria for Adverse Events
EudraCT: European Union Drug Regulating Authorities Clinical Trials registration number
NCT: Clinical trials registration number
PFS: Progression free survival
OS: Overall survival
FOLFOX: 5-Fluorouracil, Leucovorin, Oxaliplatin
DNA: Deoxyribonucleic acid
mCRC: Metastatic colorectal cancer
5-FU: 5-Fluorouracil
CPT-11: Irinotecan
HR-QoL: Health-related quality of life
EORTC: European Organisation for Research and Treatment of Cancer
EORTC QLQ-C30: Questionnare developed to assess the quality of life of cancer patients
EQ-5D-5L: European Quality of Life 5 Dimensions 5 Level Version
cfDNA: Cell free deoxyribonucleic acid
miRNA: Micro ribonucleic acid
IncRNA: Long non-coding ribonucleic acid
IDH1,2: Isocitrate dehydrogenase 1,2
SMAD4: Mothers against decapentaplegic homolog 4
qPCR: Quantitative polymerase chain reaction
FACS: Fluorescence-activated cell sorting
suPAR: Soluble urokinase plasminogen activator receptor
APRIL: A proliferation-inducing ligand
TWEAK: Tumor necrosis factor-like weak inducer of apoptosis
TRAIL: Tumor necrosis factor-related apoptosis-inducing ligand
CCL: CC-chemokine ligand
CXCR: C-X-C chemokine receptor
16S RNA: 16S ribonucleic acid
EU: European union
ECOG: Eastern Cooperative Oncology Group
ANC: Absolute neutrophil count
ULN: Upper limit of normal
INR: International normalized ratio
PTT: Partial thromboplastin time
AST: Aspartate Aminotransferase
SGOT: Glutamic-oxaloacetic transaminase
ALT: Alanine Aminotransferase
SGPT: Serum glutamic pyruvic transaminase
CNS: Central nervous system
TACE: Transarterial chemoembolization
RFA: Radiofrequencyablation
SIRT: Selective intraarterial radiotherapy
AMG: German Medicine Act
MRI: Magnetic resonance imaging
CT: Computed tomography
BSA: Body surface area
BID: Bis in die
eCRF: Electronic case reporting form
5-HT3: 5-Hydroxytryptamine
G-CSF: Granulocyte colony-stimulating factor
SmPC: Summary of Product Characteristics
KKSD: Coordination Center for Clinical Trials Düsseldorf
(S)AE: (Severe) adverse event
AE: Adverse event
SAE: Severe adverse event
SADR: Serious adverse drug reaction
QoL: Quality of Life
VAS: Visual analogue scale
ICH: International Council for Harmonisation of Technical Requirements for Pharmaceuticals for Human Use
GCP: Good Clinical Practice
FOLFIRI: 5-Fluorouracil, Leucovorin, Irinotecan
FUFIRI: Irinotecan, 5-fluorouracil, leucovorin

## References

[1] Banales JM, Marin JJG, Lamarca A, Rodrigues PM, Khan SA, Roberts LR, Cardinale V, Carpino G, Andersen JB, Braconi C, Calvisi DF, Perugorria MJ, Fabris L, Boulter L, Macias RIR, Gaudio E, Alvaro D, Gradilone SA, Strazzabosco M, Marzioni M, Coulouarn C, Fouassier L, Raggi C, Invernizzi P, Mertens JC, Moncsek A, Rizvi S, Heimbach J, Koerkamp BG, Bruix J, Forner A, Bridgewater J, Valle JW, Gores GJ (2020). Cholangiocarcinoma 2020: the next horizon in mechanisms and management. Nat Rev Gastroenterol Hepatol.

[2] Plentz RR, Malek NP (2015). Clinical presentation, risk factors and staging systems of cholangiocarcinoma. Best Pract Res Clin Gastroenterol.

[3] Bergquist A, von Seth E (2015). Epidemiology of cholangiocarcinoma. Best Pract Res Clin Gastroenterol.

[4] Weigt J, Malfertheiner P (2010). Cisplatin plus gemcitabine versus gemcitabine for biliary tract cancer. Expert Rev Gastroenterol Hepatol.

[5] Oh DY, He AR, Qin S, Chen LT, Okusaka T, Vogel A, Kim JW, Suksombooncharoen T, Lee MA, Kitano M, Burris HA, Bouattour M, Tanasanvimon S, Zaucha R, Avallone A, Cundom J, Rokutanda N, Xiong J, Cohen G, Valle JW (2022). A phase 3 randomized, double-blind, placebo-controlled study of durvalumab in combination with gemcitabine plus cisplatin (GemCis) in patients (pts) with advanced biliary tract cancer (BTC): TOPAZ-1. J Clin Oncol.

[6] Walter T, Horgan AM, McNamara M, McKeever L, Min T, Hedley D, Serra S (2013). Feasibility and benefits of second-line chemotherapy in advanced biliary tract cancer: a large retrospective study. Eur J Cancer.

[7] Valle J, Wasan H, Palmer DH, Cunningham D, Anthoney A, Maraveyas A, Madhusudan S (2010). Cisplatin plus gemcitabine versus gemcitabine for biliary tract cancer. N Engl J Med.

[8] Lamarca A, Hubner RA, David Ryder W, Valle JW (2014). Second-line chemotherapy in advanced biliary cancer: a systematic review. Ann Oncol.

[9] Lamarca A, Palmer DH, Wasan HS, Ross PJ, Ma YT, Arora A, Falk S, Gillmore R, Wadsley J, Patel K, Anthoney A, Maraveyas A, Iveson T, Waters JS, Hobbs C, Barber S, Ryder WD, Ramage J, Davies LM, Bridgewater JA, Valle JW, Advanced Biliary Cancer Working Group (2021). Second-line FOLFOX chemotherapy (Shitara)versus active symptom control for advanced biliary tract cancer (ABC-06): a phase 3, open-label, randomised, controlled trial. Lancet Oncol.

[10] Yoo C, Kim KP, Jeong JH, Kim I, Kang MJ, Cheon J, Kang BW, Ryu H, Lee JS, Kim KW, Abou-Alfa GK, Ryoo BY (2021). Liposomal irinotecan plus fluorouracil and leucovorin versus fluorouracil and leucovorin for metastatic biliary tract cancer after progression on gemcitabine plus cisplatin (NIFTY): a multicentre, open-label, randomised, phase 2b study. Lancet Oncol.

[11] Lamarca A, Edeline J, Goyal L (2022). How I treat biliary tract cancer. ESMO Open.

[12] Burness CB, Duggan ST (2016). Trifluridine/Tipiracil: A Review in Metastatic Colorectal Cancer. Drugs.

[13] Mayer RJ, Van Cutsem E, Falcone A, Yoshino T, Garcia-Carbonero R, Mizunuma N, Yamazaki K, Shimada Y, Tabernero J, Komatsu Y, Sobrero A, Boucher E, Peeters M, Tran B, Lenz HJ, Zaniboni A, Hochster H, Cleary JM, Prenen H, Benedetti F, Mizuguchi H, Makris L, Ito M, Ohtsu A, RECOURSE Study Group (2015). Randomized trial of TAS-102 for refractory metastatic colorectal cancer. N Engl J Med.

[14] Shitara K, Doi T, Dvorkin M, Mansoor W, Arkenau HT, Prokharau A, Alsina M, Ghidini M, Faustino C, Gorbunova V, Zhavrid E, Nishikawa K, Hosokawa A, Yalçın Ş, Fujitani K, Beretta GD, Cutsem EV, Winkler RE, Makris L, Ilson DH, Tabernero J (2018). Trifluridine/tipiracil versus placebo in patients with heavily pretreated metastatic gastric cancer (TAGS): a randomised, double-blind, placebo-controlled, phase 3 trial. Lancet Oncol.

[15] Emura T, Murakami Y, Nakagawa F, Fukushima M, Kitazato K (2004). A novel antimetabolite, Trifluridine/Tipiracil retains its effect on FU-related resistant cancer cells. Int J Mol Med.

[16] Chakrabarti S, Zemla TJ, Ahn DH, Ou FS, Fruth B, Borad MJ, Hartgers ML, Wessling J, Walkes RL, Alberts SR, McWilliams RR, Liu MC, Durgin LM, Bekaii-Saab TS, Mahipal A (2020). Phase II Trial of Trifluridine/Tipiracil in Patients with Advanced, Refractory Biliary Tract Carcinoma. Oncologist.

[17] Harsha Tella S, Wessling J, Nathan F (2022). CLO22–088: Phase II Trial of Trifluridine/Tipiracil in Combination With Irinotecan in Advanced Biliary Tract Cancers (BTCs). J Natl Compr Canc Netw.

[18] Choi IS, Kim KH, Lee JH, Suh KJ, Kim JW, Park JH, Kim YJ, Kim JS, Kim JH, Kim JW (2021). A randomised phase II study of oxaliplatin/5-FU (mFOLFOX) versus irinotecan/5-FU (mFOLFIRI) chemotherapy in locally advanced or metastatic biliary tract cancer refractory to first-line gemcitabine/cisplatin chemotherapy. Eur J Cancer.

[19] Eisenhauer EA, Therasse P, Bogaerts J, Schwartz LH, Sargent D, Ford R, Dancey J (2009). New response evaluation criteria in solid tumors: revised RECIST guideline (version 1.1). Eur J Cancer.

[20] Varghese AM, Cardin DB, Hersch J, Benson AB, Hochster HS, Makris L, Hamada K, Berlin JD, Saltz LB (2020). Phase I Study of Trifluridine/Tipiracil Plus Irinotecan and Bevacizumab in Advanced Gastrointestinal Tumors. Clin Cancer Res.

[21] Moretto R, Raimondo L, De Stefano A, Cella CA, Matano E, De Placido S, Carlomagno C (2013). FOLFIRI in patients with locally advanced or metastatic pancreatic or biliary tract carcinoma. Anti-Cancer Drugs.

[22] Turnbull BW (1976). The empirical distribution function with arbitrarily grouped, censored and truncated data. J R Stat Soc Ser B.

[23] Newcombe RG (1998). Two-sided confidence intervals for the single proportion: comparison of seven methods. Stat Med.

[24] Schwarz R, Hinz A (2001). Reference data for the quality of life questionnaire EORTC QLQ-C30 in the general German population. Eur J Cancer.

[25] Aaronson NK, Ahmedzai S, Bergman B, Bullinger M, Cull A, Duez NJ, Filiberti A (1993). The European Organization for Research and Treatment of Cancer QLQ-C30: a quality-of-life instrument for use in international clinical trials in oncology. J Natl Cancer Inst.

[26] King MT (1996). The interpretation of scores from the EORTC quality of life questionnaire QLQ-C30. Qual Life Res.

[27] Hjermstad MJ, Fayers PM, Bjordal K, Kaasa S (1998). Using reference data on quality of life–the importance of adjusting for age and gender, exemplified by the EORTC QLQ-C30 (+3). Eur J Cancer.

[28] Uwer L, Rotonda C, Guillemin F, Miny J, Kaminsky MC, Mercier M, Tournier-Rangeard L (2011). Responsiveness of EORTC QLQ-C30, QLQ-CR38 and FACT-C quality of life questionnaires in patients with colorectal cancer. Health Qual Life Outcomes.

[29] Pickard AS, Wilke CT, Lin HW, Lloyd A (2007). Health utilities using the EQ-5D in studies of cancer. Pharmacoeconomics.

[30] Okusaka T, Nakachi K, Fukutomi A, Mizuno N, Ohkawa S, Funakoshi A, Nagino M (2010). Gemcitabine alone or in combination with cisplatin in patients with biliary tract cancer: a comparative multicentre study in Japan. Br J Cancer.

[31] Lamarca A, Palmer DH, Wasan HS, Ross PJ, Ma YT, Arora A, Falk S, Gillmore R, Wadsley J, Patel K, Anthoney A, Maraveyas A, Iveson T, Waters JS, Hobbs C, Barber S, Ryder WD, Ramage J, Davies LM, Bridgewater JA, Valle JW, Advanced Biliary Cancer Working Group (2021). Second-line FOLFOX chemotherapy versus active symptom control for advanced biliary tract cancer (ABC-06): a phase 3, open-label, randomised, controlled trial. Lancet Oncol..

[32] Brieau B, Dahan L, De Rycke Y, Boussaha T, Vasseur P, Tougeron D, Lecomte T, Coriat R, Bachet JB, Claudez P, Zaanan A, Soibinet P, Desrame J, Thirot-Bidault A, Trouilloud I, Mary F, Marthey L, Taieb J, Cacheux W, Lièvre A (2015). Second-line chemotherapy for advanced biliary tract cancer after failure of the gemcitabine-platinum combination: a large multicenter study by the Association des Gastro-Entérologues Oncologues. Cancer.

[33] Sasaki T, Isayama H, Nakai Y, Takahara N, Satoh Y, Takai D, Kogure H, Yamamoto N, Hirano K, Tada M, Yatomi Y, Koike K (2013). A pilot study of salvage irinotecan monotherapy for advanced biliary tract cancer. Anticancer Res.

[34] Feisthammel J, Schoppmeyer K, Mössner J, Schulze M, Caca K, Wiedmann M (2007). Irinotecan with 5-FU/FA in advanced biliary tract adenocarcinomas: a multicenter phase II trial. Am J Clin Oncol.

[35] Guion-Dusserre JF, Lorgis V, Vincent J, Bengrine L, Ghiringhelli F (2015). FOLFIRI plus bevacizumab as a second-line therapy for metastatic intrahepatic cholangiocarcinoma. World J Gastroenterol.

[36] Wang-Gillam A, Li CP, Bodoky G, Dean A, Shan YS, Jameson G, Macarulla T, Lee KH, Cunningham D, Blanc JF, Hubner RA, Chiu CF, Schwartsmann G, Siveke JT, Braiteh F, Moyo V, Belanger B, Dhindsa N, Bayever E, Von Hoff DD, Chen LT, NAPOLI-1 Study Group (2016). Nanoliposomal irinotecan with fluorouracil and folinic acid in metastatic pancreatic cancer after previous gemcitabine-based therapy (NAPOLI-1): a global, randomised, open-label, phase 3 trial. Lancet.

[37] Petrelli F, Inno A, Ghidini A, Rimassa L, Tomasello G, Labianca R, Barni S (2017). Second line with oxaliplatin- or irinotecan-based chemotherapy for gemcitabine-pretreated pancreatic cancer: a systematic review. Eur J Cancer.

[38] SmPC Lonsurf®. Date of revision: January 2021. Les Laboratoires Servier, 92284 Suresnes Cedex, France, Marketing authorization number: EU/ 1/16/1096/001 – 006.

[39] Uboha N, Hochster HS (2016). TAS-102: a novel antimetabolite for the 21st century. Future Oncol.

[40] De Falco V, Napolitano S, Roselló S (2020). How we treat metastatic colorectal cancer. ESMO Open.

[41] Lenz HJ, Stintzing S, Loupakis F (2015). TAS-102, a novel antitumor agent: a review of the mechanism of action. Cancer Treat Rev.

[42] Doi T, Yoshino T, Fuse N, Boku N, Yamazaki K, Koizumi W, Shimada K, Takinishi Y, Ohtsu A (2015). Phase I study of TAS-102 and irinotecan combination therapy in Japanese patients with advanced colorectal cancer. Invest New Drugs.

[43] Hara H, Mizukami T, Minashi K, Nishina T (2021). A pase I/II trial of trifluridine/tipiracil in combination with irinotecan in patients with advanced gastric cancer refractory to fluoropyrimidine, platinum an taxane. J Clin Oncol.

